# Chronic Pelvic Puzzle: Navigating Deep Endometriosis with Renal Complications

**DOI:** 10.3390/jcm13010220

**Published:** 2023-12-30

**Authors:** Ileana Adela Vacaroiu, Andra-Elena Balcangiu-Stroescu, Iulia-Ioana Stanescu-Spinu, Daniela Gabriela Balan, Mihai-Teodor Georgescu, Maria Greabu, Daniela Miricescu, Elena Cuiban, Larisa Florina Șerban-Feier, Mircea Ovidiu Denis Lupușoru, Alexandra Gaube, Dragos-Eugen Georgescu

**Affiliations:** 1 Department of Nephrology, Faculty of Medicine, Carol Davila University of Medicine and Pharmacy, 020021 Bucharest, Romania; ileana.vacaroiu@umfcd.ro (I.A.V.); elena.cuiban@drd.umfcd.ro (E.C.); larisa-florina.feier@drd.umfcd.ro (L.F.Ș.-F.); 2 Department of Physiology, Faculty of Dentistry, Carol Davila University of Medicine and Pharmacy, 050474 Bucharest, Romania; andra.balcangiu@umfcd.ro (A.-E.B.-S.); iulia.stanescu@umfcd.ro (I.-I.S.-S.); 3 “Prof. Dr. Al. Trestioreanu” Oncology Discipline, Faculty of Medicine, Carol Davila University of Medicine and Pharmacy, 020021 Bucharest, Romania; 4 Department of Biochemistry, Faculty of Dentistry, Carol Davila University of Medicine and Pharmacy, 8 Eroii Sanitari Blvd, 050474 Bucharest, Romania; maria.greabu@umfcd.ro (M.G.); daniela.miricescu@umfcd.ro (D.M.); 5 Department of Physiology I, Faculty of Medicine, Carol Davila University of Medicine and Pharmacy, 8 Eroii Sanitari Blvd, 050474 Bucharest, Romania; mircea.lupusoru@umfcd.ro; 6 National Institute of Infectious Diseases “Prof. Dr. Matei Bals”, 1st Doctor Calistrat Grozovici St., 021105 Bucharest, Romania; alexandra.gaube@rez.umfcd.ro; 7 “Dr. Ion Cantacuzino” General Surgery Discipline, Faculty of Medicine, Carol Davila University of Medicine and Pharmacy, 8 Eroii Sanitari Blvd, 050474 Bucharest, Romania; dragos-eugen.georgescu@umfcd.ro

**Keywords:** endometriosis, renal complications, chronic pelvic pain, surgery

## Abstract

This case report delves into the intricacies of a challenging clinical scenario involving deep pelvic endometriosis, which manifested with renal complications. Endometriosis, a complex gynecological condition, is explored in this case, highlighting its multifaceted nature. The patient presented with a complex interplay of symptoms, including chronic pelvic pain, urinary tract issues, and severe deep adenomyosis. The diagnostic journey was protracted, emphasizing the need for early recognition and intervention in such cases. A thorough evaluation, including laparoscopic examination and histopathological analysis, revealed the extensive presence of endometriotic lesions in various pelvic and renal structures, ultimately leading to left hydronephrosis. The report underscores the significance of timely diagnosis and surgical intervention to prevent irreversible renal damage. This case provides valuable insights into the management of deep endometriosis with renal involvement and the importance of interdisciplinary collaboration. Understanding the complexities of this condition can aid in improving patient outcomes and enhancing the quality of care provided.

## 1. Introduction

Endometriosis is a clinically important and often underdiagnosed condition, characterized by the localization and growth of endometrial tissue outside the uterine cavity, accompanied by chronic inflammation [1,2,3]. The most common sites for ectopic endometrial implants are the ovaries, ovarian fossa, uterosacral ligaments, and the posterior cul-de-sac [3]. It can manifest as infiltrative, deep, or superficial lesions on the peritoneum and serosa [1]. Additionally, endometriosis can potentially transform into a malignant condition, with a 0.7–1.6% chance of progressing to clear cell carcinoma or endometrioid carcinoma [4]. In such cases, treatment becomes more complex and may involve surgical procedures, radiotherapy, immunotherapy, and algorithms for preventing complications like tumor lysis syndrome and acute kidney injury [5].

The prevalence of this condition increases up to 30% in patients with infertility and up to 45% in patients with chronic pelvic pain [1,6]. It is a common pelvic pathology in females, affecting an estimated 15% of reproductive-age women [7,8,9]. Endometriosis is a debilitating disease with various severe effects on social, occupational, and psychological functioning [10]. It shares some similarities with malignant conditions, such as progressive and invasive growth, estrogen dependence, recurrence, and a tendency to metastasize [11,12]. Genetically, genomic regions and anomalies in pro-cancer genes (PIK3CA, KRAS, and ARID1A) have been associated with endometriosis [13,14,15]. The presence of pro-cancer mutations in non-malignant cells can partially explain the aggressive nature of deeply invasive lesions compared to superficial peritoneal lesions.

Endometriosis is classified based on the severity of symptoms, affected areas, location, depth (infiltration more than 5 mm below the peritoneum), and growth rate into four stages: Stage I (minimal disease), Stage II (mild disease), Stage III (moderate disease), and Stage IV (severe disease) [16,17]. However, this classification does not always predict clinical outcomes, including symptoms and pain [10].

Other sources of ectopic endometrial cells include the mesothelium, stem cells, Müllerian remnants, bone marrow stem cells, embryonic remnants, and lymphatic or vascular dissemination, as well as celomic metaplasia [18,19]. The hypothesis of retrograde menstruation is questioned by the existence of endometriosis in girls before their first menstruation, implying the involvement of embryonic Müllerian remnants [20,21]. These premenarchal lesions are considered preexisting forms of classical endometriosis and result from neonatal uterine bleeding, including retrograde bleeding, due to exposure to maternal hormones [22,23].

Diagnosing this disease remains challenging because many patients are asymptomatic, and the definitive diagnosis often involves surgical procedures. In recent years, progress has been made in identifying biomarkers that may correlate with a positive diagnosis of endometriosis, including the soluble fraction of urokinase-type plasminogen activator receptor, which is involved in various other pathologies [24,25].

Symptoms of endometriosis depend on the location of the endometriotic lesions. Chronic pelvic pain, often related to menstruation, dyspareunia, and dysuria are common manifestations, but they can also be seen in other conditions. In endometriosis, inflammatory changes occur due to the increased production of inflammatory mediators that trigger pain [26,27,28,29,30].

This pathology predominantly affects the pelvic reproductive organs but can also be found in non-reproductive organs, referred to as extragenital endometriosis [2]. The most common locations for its development, both genital and extragenital, are, in descending order, the ovary and ovarian fossa (67% and 32%), anterior and posterior cul-de-sac, late posterior ligaments, uterosacral ligaments (46%), Douglas pouch (30%), bladder (21%), uterus, uterine tubes, sigmoid colon, appendix, and round ligaments. Other rare sites for endometriosis have been reported in the breast, pancreas, liver, gallbladder, kidney, urethra, extremities, vertebrae, bone, peripheral nerves, spleen, diaphragm, central nervous system, hymen, and lungs [31].

Extragenital lesions are more commonly diagnosed between the ages of 35 and 40 and are usually discovered later than genital lesions, approximately 5 years after their appearance [32]. A small percentage of endometriosis cases (0.1–1%) occur in the urinary tract, often underestimated due to the late diagnosis of subclinical progression [7].

Bladder endometriosis is typically located on the posterior wall or dome, less frequently involving the base of the bladder, and is proximal to the ureteral orifice. Ureteral endometriosis can be intrinsic or extrinsic, with a 1:4 ratio, and the ureter is usually involved below the pelvic brim [33,34]. Ureterohydronephrosis is a consequence of urinary tract obstruction and can affect a single ureter, more commonly the left one [35], or both ureters, especially in patients with extensive pelvic endometriosis. Ureteral endometriosis is typically discovered in women aged 30 to 35 years [36,37,38], with a lower frequency in postmenopausal women [39].

In the extrinsic form (75% of cases), ureteral endometriosis is localized in the adventitia or around the connective tissue of the ureter. In intrinsic endometriosis, endometriotic tissue infiltrates the muscular wall of the ureter. In about one-third of cases, urinary stasis caused by hydronephrosis promotes the development of urinary tract infections, especially upper urinary tract infections [40]. Symptoms related to ureteral endometriosis are often nonspecific, and severe stenosis can lead to symptomatic hydronephrosis and ultimately a decline in renal function [41].

Rare cases have been reported of patients with chronic kidney disease based on obstructive nephropathy caused by bilateral ureteral obstruction, but the incidence of CKD due to endometriosis is unknown. The risk of silent renal loss in these patients is reported to be as high as 25–50% [42,43,44].

The aim of this paper is to explore a challenging clinical scenario characterized by extensive pelvic endometriosis, leading to renal complications. Emphasizing the critical role of prompt diagnosis and surgical intervention in avoiding irreversible renal impairment, it offers crucial insights into our course of action regarding deep endometriosis accompanied by renal implications. Moreover, the essentiality of interdisciplinary cooperation in handling such intricate clinical cases is highlighted.

## 2. Case Presentation

We report the case of a 48-year-old female patient with no remarkable personal pathological background, with menarche at 12 years old, with a surgical history that included two cesarean deliveries, who initially presented with symptoms suggestive of cystitis, including dysuria, polyuria, and urgency. A recent outpatient ultrasound detected Grade II hydronephrosis in the left kidney. Subsequent reevaluation by ultrasound did not reveal any obstructive causes for the hydronephrosis. Considering the presenting symptoms and left-sided hydronephrosis, renal calculi with migrating stones were suspected.

A series of imaging investigations were conducted to establish a correct diagnosis and, consequently, determine the appropriate treatment. The next imaging investigation involved a contrast-enhanced abdominal CT scan, confirming Grade II left-sided hydronephrosis, with a possible cause being a low-level stenosis. It also described the uterus with a dense image adhered to the posterior wall, possibly indicating hematoma or pregnancy.

Based on the CT results, an interdisciplinary nephro-urological report recommended gynecological consultation with subsequent urological assessment and left ureteral drainage with a JJ stent. Two gynecological consultations, which indicated no pathological findings during the clinical examination, were succeeded by transvaginal ultrasounds, highlighting significant changes in the uterine body and ovaries, with a recommendation for pelvic MRI.

This imaging assessment did not reveal any apparent obstructive etiologies for urinary system disorders. However, it confirmed the existence of anatomical alterations within the reproductive system, including moderate changes indicative of homogeneous diffuse adenomyosis on the posterior uterine wall. Additionally, signal modifications were evident in the outer one-third of the myometrium along the posterior corporeal uterine slope, suggesting the presence of a focal adenomyosis area, concomitant with a small uterine leiomyoma. Following this, the patient sought a urological outpatient consultation, which addressed the suspicion of deep endometriosis and uretero-vesical junctional syndrome. It is noteworthy to highlight that abnormalities in the reproductive tract were the only risk factors associated with endometriosis identified in this patient’s case.

**Figure 1:**
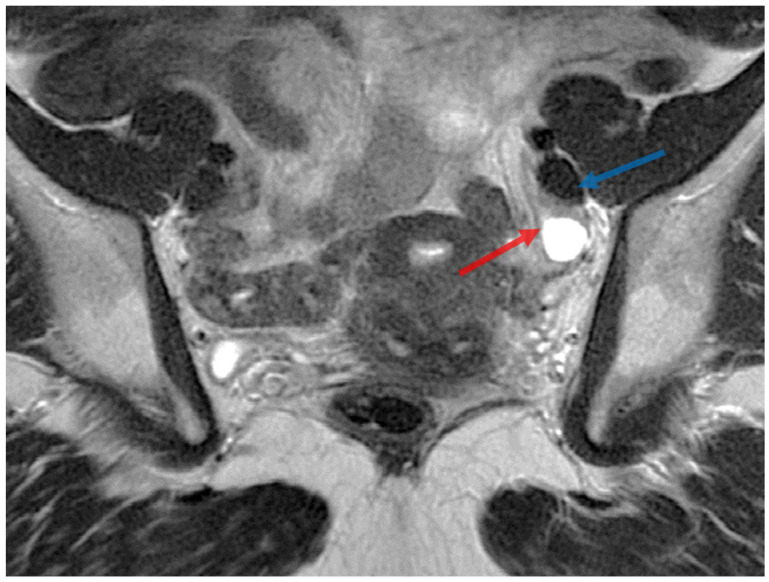
Coronal T2 section. The endometriosis nodule (red arrow), isointense on T2, with an unclear demarcation, located in the left pelvic area, closely related to the ureter. Simple left ovarian cyst (blue arrow).

Approximately two months later, the patient underwent a subsequent pelvic MRI. The results displayed a retroflexed uterus, slightly laterally deviated to the left, with a maximum transverse diameter of 5.3 cm and a longitudinal diameter of 10 cm. The endometrium had a normal thickness of 8 mm with a homogeneous signal, while the myo-endometrial junction appeared diffusely thickened (up to 14 mm), with an irregular contour towards the rest of the myometrium and included T2 hyperintense spots. There were intramural leiomyomas, measuring 10/12 mm and 8/14 mm on the left lateral aspect of the uterine body and 9/10 mm on the posterior aspect of the uterine body. A small intracavitary leiomyoma was observed on the right lateral aspect of the uterine body, measuring 5/7 mm. A post-cesarean uterine scar with diverticular dilation was present at this level (8/14 mm). On the right side, an ovarian endometrioma, measuring 8/9 mm medially, and a newly developed right ovarian cyst, measuring 27/35 mm, with hemorrhagic content, were observed. The cyst did not exhibit characteristics of an endometrioma (hemorrhagic cyst—requiring ultrasound monitoring). There were no ovarian endometriomas on the left side, and no pathological tubal accumulations were found bilaterally. A large nodule of deep endometriosis was detected on the posterior uterine wall (Figure 1), in the body region, encompassing the attachment of the utero-sacral ligaments and retrocervical area. It had a maximum transverse diameter of approximately 50 mm, antero-posterior diameter of 30 mm, and a longitudinal diameter of 35 mm. The nodule showed infiltration into the myometrium and adhesion to the posterior vaginal fornix and upper rectal serosa (over a distance of approximately 10 mm, around 10 cm cranial to the external anal orifice).

**Figure 2:**
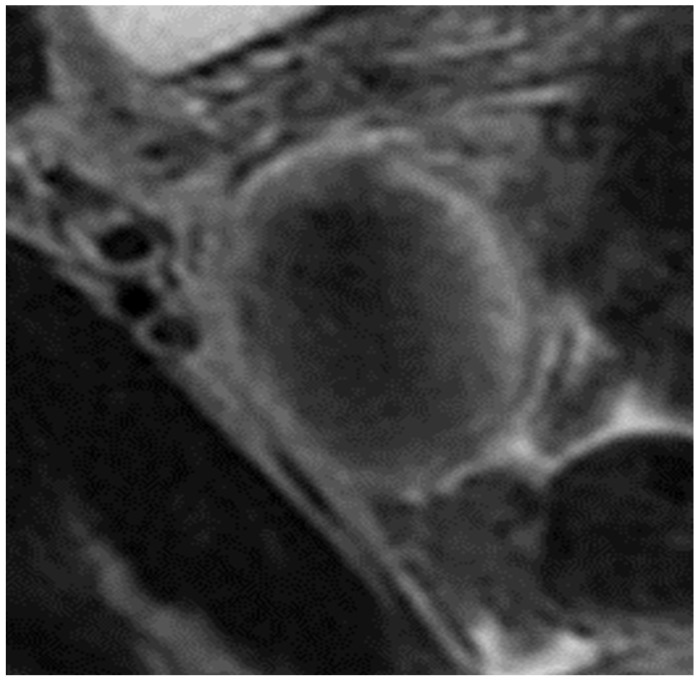
Coronal T2 fat-saturated section. Ovarian endometrioma located on the medial side of the right adnexa, displaying a characteristic “shading sign” (a radiological hallmark of endometriosis due to high protein and iron content secondary to hemorrhage).

Nodular infiltration was observed along the course of the utero-sacral ligaments and bilateral parametrial regions, with more significant involvement on the left side and bilateral ovarian adhesions (Figure 2). A left parametrial nodule, measuring approximately 20/40 mm, encompassed and stenosed the left pelvic ureter over a length of approximately 15 mm, at around 25 mm cranial to the left ureteral orifice. A left pelvic ureteral dilation upstream of the stenotic area was observed, with a maximum diameter of 12 mm. No deep nodules were detected in the sigmoid colon, bladder, or the anterior abdominal wall. The right pelvic ureter remained nondilated, and there were minimal pelvic ascites with a maximum thickness of 24 mm in the Douglas space.

Following extensive multidisciplinary assessment and a series of imaging evaluations, the patient was admitted to our specialized endometriosis center for comprehensive disease management.

The interdisciplinary team opted for surgical intervention through a laparoscopic approach to conduct the biopsy of multiple endometriosis nodules, perform a left ureter dissection followed by reimplantation, and undertake a radical hysterectomy with bilateral adnexectomy.

The exploratory laparoscopy revealed a complex pelvic adhesive syndrome involving the small bowel loops, a significantly enlarged uterus (severe deep adenomyosis), both adnexa (right ovarian endometrioma—20 mm), sigmoid colon, rectosigmoid junction, upper rectum, and both ureters (left ureter dilated) with extensive left parametrial infiltration. Viscerolysis was performed with difficulty, including dissection of the sigmoid colon, upper rectum, rectovaginal space, prevesical space, and both ureters, as well as bilateral ovarian suspension. Several excisions of endometriosis nodules were performed, including the ureteral nodule: extensive endometriotic lesions in the retro-uterine and retrocervical regions; an endometriotic nodule in the right parametrium and right uterosacral ligament—excision with ureteral dissection; a voluminous ureteral endometriotic nodule (extrinsic endometriosis); resection of the left ureter with vesicoureteral anastomosis; catheterization of the left ureter; and a voluminous endometriotic nodule in the left parametrium, in the vicinity of the sacral roots.

### Histopathological Examination Results

**Figure 3:**
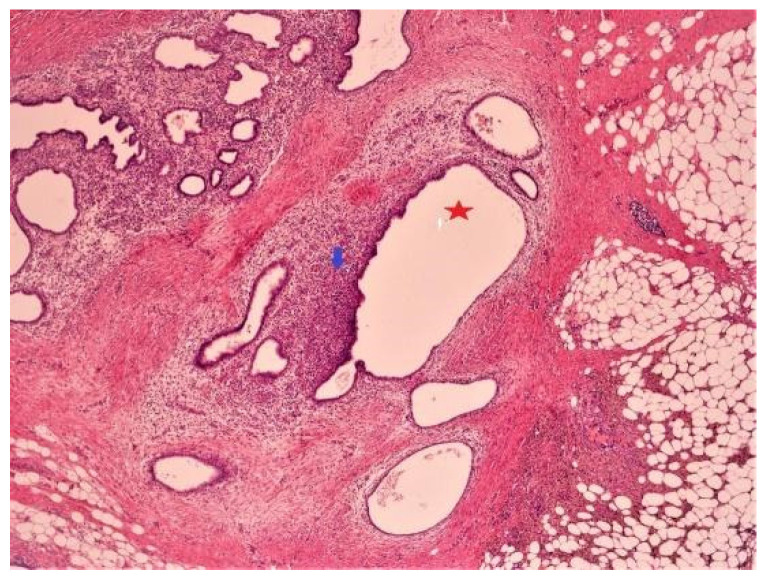
Pseudotumoral formation of the left ureteral nodule: fibroadipose tissue with ectopic endometrial glands (red star) and endometrial stroma (blue arrow).

Examination of the “left ureteral nodule” specimen: the histopathological appearance indicates the presence of endometriotic lesions (Figure 3).

Examination of the “uterus + cervix + fallopian tubes” specimen: Chronic papillary endocervicitis. Uterine leiomyomas. Superficial and deep adenomyosis. Secretory endometrium. Simple serous paratubal cysts. Histopathological appearance suggestive of tubal endometriotic lesions.

**Figure 4:**
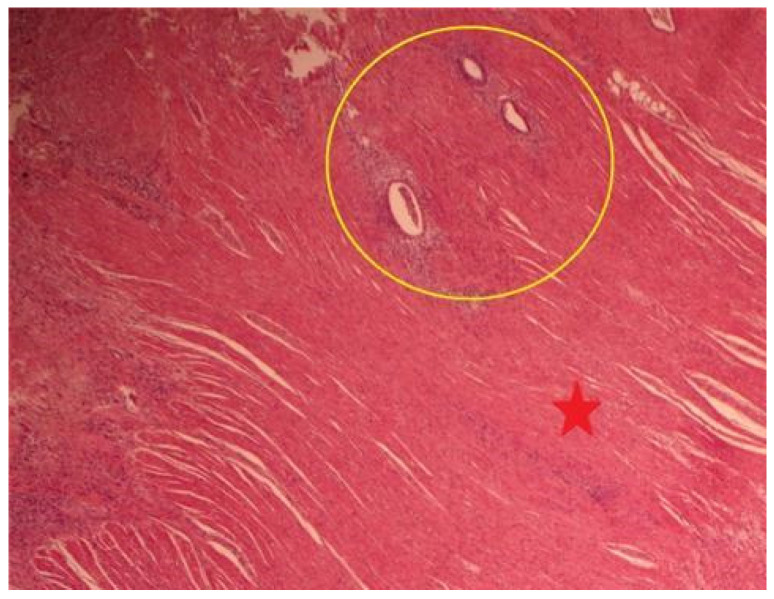
Submucosa and muscularis propria (red star) with foci of endometriosis (yellow circle).

Examination of the “endometriotic lesions” specimen, which includes two tissue fragments: The histopathological appearance indicates the presence of endometriotic lesions (Figure 4).

The patient’s post-operative course was favorable. Three weeks later, she presented for the removal of the left JJ stent and subsequently had a good general condition, was afebrile, and had clear urine.

The entire process from presentation to the nephrology department to the final diagnosis and surgical treatment took three months.

Laboratory tests at three months showed no changes, and abdominal ultrasound of the left kidney revealed a structurally normal kidney without hydronephrosis. The patient received recommendations for periodic medical follow-up [45]

## 3. Discussion

In the context of endometriosis with ureteral invasion, understanding the diverse anatomical locations of endometriotic lesions becomes crucial for comprehensive management. Notably, studies have highlighted the multifaceted nature of endometriosis, implicating varied sites of involvement. Habib et al. provided a comprehensive perspective on bowel endometriosis, underscoring diagnostic and therapeutic aspects [46]. Gustofson et al. conducted a case series and comprehensive review, emphasizing the association between endometriosis and the appendix [47]. Furthermore, insights from Jenkins et al. and Audebert et al. underscored the significance of the anatomic distribution of endometriosis lesions, shedding light on its pathogenetic implications [48,49]. Additionally, rare occurrences such as Villar’s nodule and umbilical endometriosis, as documented by Victory et al., Jaime et al., and Lee et al., further exemplify the diverse and atypical locations in which endometriotic lesions can manifest [50,51,52]. Understanding these varied sites of endometriosis manifestation is pivotal in diagnosing and managing cases involving unusual presentations, such as ureteral invasion, providing a comprehensive approach to treatment strategies.

Deep infiltrating endometriosis is defined as lesions penetrating surrounding tissue by 5 mm or more and is likely of multifactorial origin [53]. Endometriosis is a common disorder affecting women of all ages, that can lead to depression and anxiety disorders, with a decrease in workability, restrictions in social activities, and a diminished quality of life. It is estimated that 0.3 to 12% of women diagnosed with endometriosis also have urinary tract involvement. This number increases to 14 to 20% in patients with deep infiltrating endometriosis. Endometriosis can perturb the urogenital tract, in particular the ureter, which can potentially result in ureteral compression or stenosis. Even though this is rare, the consequences are dramatic, such as hydronephrosis or organ failure [45,54,55,56]. Jadoul et al. reported that the risk of loss of renal function in cases of ureteral endometriosis is 11.5% [57].

The pathogenesis of deeply infiltrative endometriosis is still a subject of study. One hypothesis is that pelvic endometriosis may be a direct extension of endometrial cells outside the uterine wall, possibly facilitated by previous pelvic surgeries [58]. This hypothesis is supported by the fact that the diagnosis of ureteral endometriosis is often preceded by hysterectomy and bilateral salpingo-oophorectomy, possibly due to prior symptoms related to adenomyosis or pelvic endometriosis. Ureteral endometriosis typically involves the lower third of the left ureter because deep infiltration of the pelvis by endometriosis is often asymmetrical and primarily affects the left pelvis, including neighboring structures such as the bladder [59].

Endometriosis of the urinary tract is an uncommon clinical finding, and it may have a slow and insidious progression, sometimes diagnosed after irreversible renal structural changes have occurred, such as unilateral or bilateral renal atrophy in an undefined number of patients. Many patients initially present to nephrologists with advanced kidney disease due to chronic urinary tract obstructions. Nephrologists need to closely monitor renal function and renal ultrasound in patients with deteriorating renal function or renal structural changes to achieve an early diagnosis of endometriosis when necessary. In patients with signs of urinary tract dilation or echogenicity abnormalities on ultrasound that sometimes associate signs of renal dysfunction, further investigations are recommended, including MRI and ureteroscopy, as well as interdisciplinary consultations [59].

The indications of urinary tract endometriosis can be elusive. In our case, the patient exhibited no apparent symptoms except for dysuria, polyuria, and urgency, resembling cystitis. Interestingly, she did not report the typical symptoms associated with endometriosis, such as dysmenorrhea or dyspareunia. Highlighting the significance for healthcare professionals managing individuals with deep infiltrating endometriosis, it is crucial to consider the potential of asymptomatic ureteral involvement. Employing imaging tests for early diagnosis and addressing any obstruction through surgical intervention is imperative to prevent the loss of renal function. Ureteral endometriosis should be considered among the potential differential diagnoses when encountering unexplained hydronephrosis in women of childbearing age. Individuals experiencing dysmenorrhea should be particularly aware of this condition [35].

The presence of chronic inflammation in endometriosis can trigger signaling pathways that lead to necrosis and a compromised immune response, heightening the susceptibility to infections, particularly in the genitourinary system. Chronic endometritis and surgical site infections become more prevalent risks. Infections involving Gardnerella, Streptococcus, Enterococci, Escherichia coli, mollicutes, and shigella have been identified as associated factors [60,61].

There is emerging evidence suggesting a shared pathway between autoimmunity and endometriosis, presenting a promising avenue for research aimed at developing immunostimulatory drugs that could demonstrate effectiveness [62]. Ongoing research endeavors focus on identifying non-invasive imaging markers for early diagnosis, aiding in the classification of cases that necessitate surgical intervention and those that could benefit from non-surgical management. This holistic approach involves a combination of pathophysiological diagnosis, imaging techniques, and laparoscopic surgeries [63].

The role of cystoscopy in diagnosis remains a subject of controversy. Ros et al. conducted a study where flexible cystoscopy was routinely performed in their institution for women exhibiting suspected bladder endometriosis affecting the muscular layer on ultrasound. They concluded that cystoscopy might not be necessary for nodules partly within the muscular layer detected via transvaginal ultrasound [64].

Despite being invasive, cystoscopy is cost-effective and aids in estimating the distances between ureteral orifices and nodule boundaries, enabling a potential biopsy. However, the intraperitoneal origin of nodules results in typical outpatient cystoscopy showing normal findings in about half of the cases, with the classic appearance of adenomatous and nodular red or bluish masses being visible only occasionally and ulcerations being rarer [64,65].

To enhance accuracy, a proposed approach involves performing cystoscopy under sedation concurrent with a pelvic examination, including bimanual palpation of the bladder. In a study of 157 participants using dynamic cystoscopy, researchers noted a high specificity (97.78%) but relatively low sensitivity (58.21%), with a significant positive predictive value (95.12%) and negative predictive value (75.86%).

Recent reviews, such as the one by Lima Diniz et al., suggest that while ultrasonography and cystoscopy serve as initial diagnostic tools, magnetic resonance imaging emerges as the most reliable method for diagnosis [66].

Generally, MRI is very useful for guiding laparoscopy, and fat-saturation MRI allows for the adequate evaluation of the location, size, and subperitoneal lesion extension of deep pelvic endometriosis, providing key information for both the diagnosis and treatment planning [67,68,69]. Our patient had endometriotic infiltration in the utero-sacral ligaments and bilateral parametrial regions, with more significant involvement on the left side, bilateral ovarian adhesions, and a left parametrial endometriosis nodule, which encompassed and stenosed the left pelvic ureter. From a prognostic standpoint, positive outcomes, especially in terms of renal function, can be achieved when both diagnosis and surgical intervention occur early, accompanied by long-term follow-up [54].

Given the infrequency of ureteral endometriosis, determining the optimal management strategies poses a challenge. For mild symptoms, conservative approaches involving nonsteroidal anti-inflammatory medications might offer sufficient relief. In more pronounced cases, medical management utilizing GnRH analogues or oral contraceptives could be considered, although hormone therapy is typically recommended for early-stage disease [33,70,71].

The prognosis of ureteral endometriosis depends on the timing of diagnosis [34]. To determine the appropriate therapy for endometriosis, the patient’s age, desire for reproduction, symptom severity, overall distribution of endometriosis, and the size of urinary tract lesions must be taken into consideration [68]. Therapy for endometriosis includes bilateral ovariectomy, as well as surgical lesion resection preceded or combined with medical castration using drugs such as danazol or gonadotropin-releasing hormone agonists [72].

The successful laparoscopic treatment of ureteral endometriosis has been described thoroughly in the literature [6,12]. Camanni and colleagues recently reviewed the surgical approach for different stages of ureteral involvement, suggesting that mild-to-moderate infiltration should be managed with ureterolysis, while cases of infiltrative endometriosis and intrinsic ureteral disease will benefit from the section of the involved segment and ureteral reimplantation—ureteroneocystostomy [73]. The main issue is that unilateral or bilateral ureteral endometriosis can be asymptomatic, and many cases are discovered incidentally during abdominal ultrasound or laparoscopy for extensive endometriosis [73].

In contemporary practice, the established approach to treating endometriosis involves the comprehensive surgical extraction of endometrial tissue, followed by conservative hormone therapy. In terms of surgical procedures, there is a preference for employing minimally invasive techniques, and laparoscopic surgery is efficacious for the treatment of deep infiltrating endometriosis [74,75,76,77,78].

In 25–43% of instances, ureteral endometriosis may lead to ureteral obstructions, potentially resulting in a loss of kidney function. Up to 47% of patients may necessitate a nephrectomy, either due to the loss of kidney function or the presence of ureteral endometriosis lesions [7]. Also, in accordance with the updated guidelines outlined by Barocas et al. (2020) [79] in the Journal of Urology, the American Urological Association (AUA) recommends office cystoscopy for individuals with a history of gross hematuria. However, for cases without gross hematuria, further testing is advised based on risk stratification. These guidelines emphasize the importance of tailored assessments for microscopic hematuria, highlighting the significance of precise diagnostic protocols in urological evaluations.

The aim of the surgical treatment is an optimal surgical and therapeutic outcome, which results in an increase of quality of life with an efficient pain treatment policy of the disease [80].

## 4. Conclusions

Deep pelvic endometriosis poses a rare and challenging diagnostic scenario due to the nonspecific nature of reported symptoms. Symptoms can range from the classic manifestations of endometriosis (most common) to less common urinary tract symptoms. In the index case, the presentation of urinary tract symptoms added complexity to the diagnosis, making it more elusive. The nonspecific symptoms associated with ureteral endometriosis can lead to misdiagnosis, potentially causing renal damage through prolonged hydronephrosis.

Maintaining a high index of suspicion and utilizing advanced imaging modalities are crucial for achieving an earlier and more accurate diagnosis, ultimately facilitating the preservation of renal function. Radiological techniques, particularly MRI, stand as the gold standard diagnostic tools for achieving a preoperative diagnosis.

In severe and recurrent cases of endometriosis where the ureter is affected and renal function is impaired, surgical intervention becomes necessary. However, if conventional treatments do not yield lasting success, there is a risk of renal function loss, and the levels of pain and discomfort can be substantial. In such challenging cases, laparoscopic ureteral reimplantation emerges as a promising therapeutic approach.

Emphasizing a heightened awareness for early diagnosis and management is crucial, as both elements play a pivotal role in achieving a better prognosis.

## Data Availability

Manuscript data are available from the corresponding author at request.
